# Progression of Prothrombin Induced by Vitamin K Absence-II in Hepatocellular Carcinoma

**DOI:** 10.3389/fonc.2021.726213

**Published:** 2021-11-10

**Authors:** Yang Yang, Guangbing Li, Ziwen Lu, Yong Liu, Junjie Kong, Jun Liu

**Affiliations:** ^1^ Department of Liver Transplantation and Hepatobiliary Surgery, Shandong Provincial Hospital, Cheeloo College of Medicine, Shandong University, Jinan, China; ^2^ Department of Liver Transplantation and Hepatobiliary Surgery, Shandong Provincial Hospital Affiliated to Shandong First Medical University, Jinan, China

**Keywords:** hepatocellular carcinoma, prothrombin induced by vitamin K absence-II, biomarker, diagnosis, prognosis

## Abstract

Hepatocellular carcinoma (HCC) is the fifth most common cancer and the third leading cause of cancer-related death worldwide. Due to the lack of efficient tools for early detection, asymptomatic HCC patients are diagnosed at an advanced stage, leading to a poor prognosis. To improve survival, serum biomarker prothrombin induced by vitamin K absence-II (PIVKA-II) was under investigation. PIVKA-II is an abnormal protein produced in HCC. The coagulation function was insufficient due to the lack of Gla residues. Elevated PIVKA-II was associated with bad tumor behavior in terms of proliferation, metastasis, and invasion. Three major signaling pathways were proposed to clarify the mechanism. With the advantages including affordability, minimal invasiveness, convenience, and efficiency, PIVKA-II could improve HCC management consisting of four aspects. First, PIVKA-II was an effective and dynamic tool for improving HCC surveillance in high-risk population. Changes in the serum levels of PIVKA-II provided valuable molecular alteration information before imaging discovery. Second, PIVKA-II offered a complementary approach for HCC early detection. Compared to traditional diagnostic approaches, the combination of PIVKA-II and other biomarkers had better performance. Third, PIVKA-II was an indicator for the assessment of response to treatment in HCC. Preoperative assessment was for selecting personalized therapy, and postoperative measurement was for assessing treatment efficacy. Fourth, PIVKA-II was considered as a prognostic predictor for HCC. Patients with elevated PIVKA-II were more likely to develop microvascular invasion, metastasis, and recurrence.

## Introduction

Hepatocellular carcinoma (HCC) is the fifth most common cancer and the third leading cause of cancer-related death worldwide (1, 2). The disease burden of HCC is increasing annually (3). The etiology of HCC is highly complex and diverse. Roughly 85% of HCC derives from cirrhosis secondary to hepatitis B virus (HBV) and hepatitis C virus (HCV) (4–6). The process in the context of chronic liver diseases takes several years or decades (7–9). Despite advancements in systemic therapies including transarterial chemoembolization (TACE), liver transplantation, targeted therapy, and immunotherapy, surgical resection is the optimal treatment for HCC. As asymptomatic HCC patients are diagnosed at an advanced stage, they are not amenable to curative surgery. The 5-year overall survival was only 15% (10–12). Thus, early detection was crucial to improving prognosis.

Currently, early detection in HCC relies on surveillance in high-risk population. Patients with chronic viral hepatitis and other chronic liver diseases such as alcoholic liver disease, non-alcoholic fatty liver disease (NAFLD), and non-alcoholic steatohepatitis (NASH) are recognized as a high-risk group of HCC. Abdominal ultrasound (US) every 6 months for populations at high risk was recommended by guidelines (13, 14). However, US is an operator-dependent technique and has limitations in the differential diagnosis. With remarkable advantages, noninvasive and cost-effective serum biomarkers were proposed. Serum α-fetoprotein (AFP) is a well-established biomarker for HCC screening. But the diagnostic accuracy is suboptimal with sensitivity at 62.4% (15–18). To address the limitations of AFP in conjunction with abdominal ultrasound, investigators proposed a novel serum biomarker for HCC surveillance and early detection—prothrombin induced by vitamin K absence-II (PIVKA-II).

PIVKA-II, also known as Des-gamma-carboxy prothrombin (DCP), is an abnormal protein produced in hepatocellular carcinoma (19). The coagulation function was insufficient due to the lack of Gla residues. Elevated PIVKA-II in HCC was associated with bad tumor behavior in terms of proliferation, metastasis, and invasion. Liebman et al. first presented PIVKA-II for HCC diagnosis in 1984 (20, 21). They found that PIVKA-II was significantly elevated in patients with HCC compared to patients with chronic liver diseases. Since then, the wide implementation of PIVKA-II had shown great performance in HCC early detection. Multiple studies suggested that PIVKA-II could facilitate HCC surveillance in high-risk population, especially for dynamic monitoring (22). Meanwhile, PIVKA-II was proposed as a potential tool for prognostic prediction in terms of vascular invasion, metastasis, and recurrence. PIVKA-II was also used in therapeutic response assessment during treatments. The efficacy of treatments including hepatic resection, liver transplantation, radiofrequency ablation, and interventional therapy could be evaluated (23–26). In this context, PIVKA-II helped guide the personalized therapies. It is worth noting that the implementation of PIVKA-II in other tumors in clinical practice has been demonstrated such as primary gastric cancer, pancreatic adenocarcinoma, etc (27, 28).

**Figure 1:**
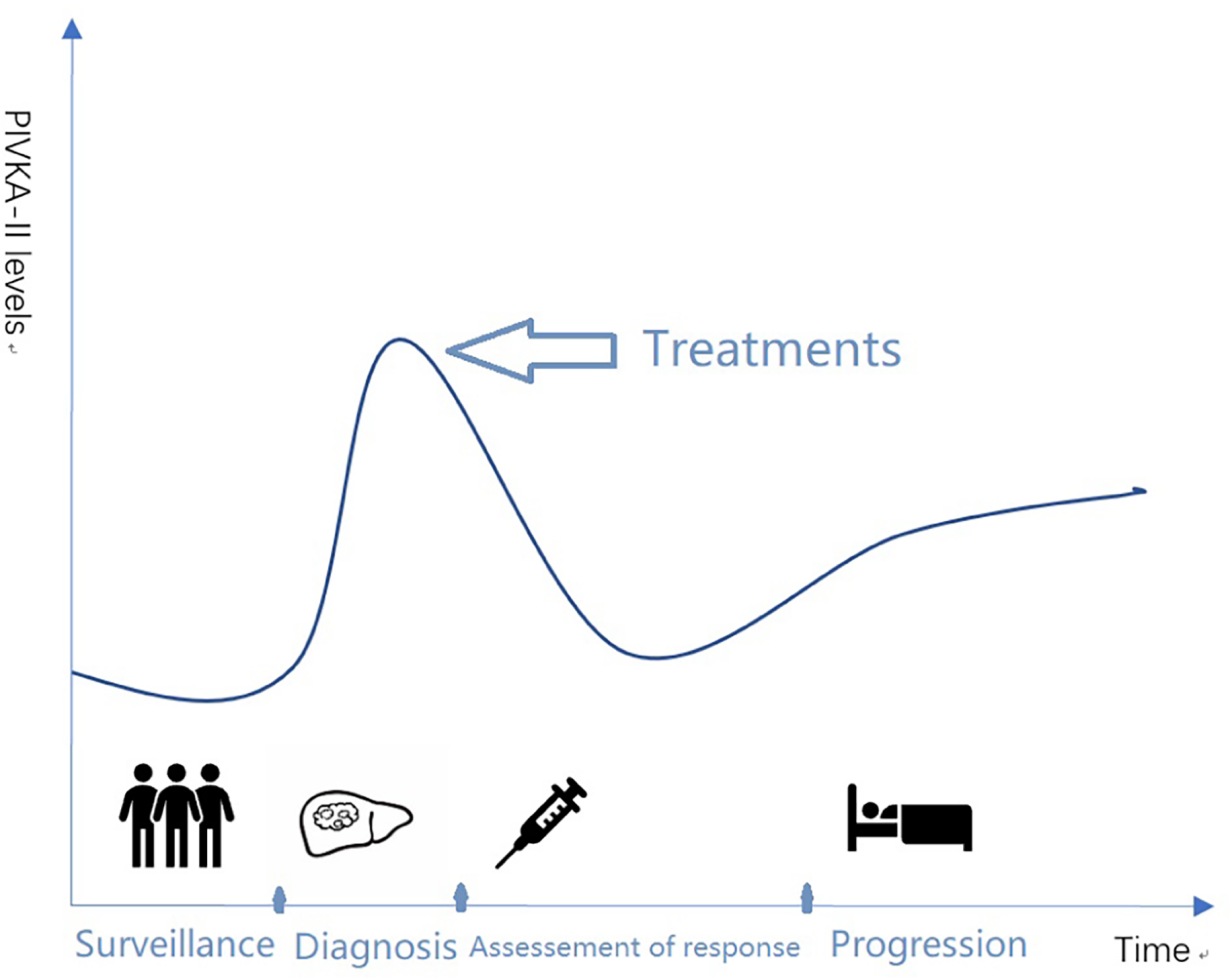
Relative levels of PIVKA-II for HCC management.

This review focused on the recent progression of PIVKA-II in HCC in the past years. The literature summarized the structure and function of PIVKA-II and clinical relevance for surveillance in high-risk population, early detection, assessment of response to therapy, and prognostic prediction in HCC (**Figure 1**).

## Structure and Signaling Pathways of PIVKA-II

### The Structural Characteristics and Biological Origin of PIVKA-II

PIVKA-II, known as Des-gamma-carboxy prothrombin (DCP), is a prothrombin precursor produced in HCC. Prothrombin in hepatocytes is constituted from amino acid residues, composed of three different structural domains, fragment 1, fragment 2, and a protease domain. There are 10 glutamic acid (Glu) residues at positions 6, 7, 14,16, 19, 20, 25, 26, 29, and 32 in the N-terminal domain (29). Normal prothrombin in hepatocyte converted the 10 Glu residues into γ-carboxylated glutamic acids termed as Gla in the protease domain (30, 31). The posttranslational carboxylation of the prothrombin precursor depended on vitamin K-dependent γ-glutamyl carboxylase. HCC cells are unable to carboxylate all of Glu residues due to carboxylase deficiency (32). The remaining Glu residues are called des-γ-carboxy prothrombin. PIVKA-II contains less than 10 Gla residues, resulting in a deficiency of thrombogen synthesis (33). Hemker et al. first observed the biosynthesis of PIVKA-II indirectly in 1963 (34). Then Nilehn and Ganrotin validated it directly (35). Stenflo et al. (36) explained the differences between PIVKA-II and normal prothrombin. They found that PIVKA-II was a kind of prothrombin lacking modified residues with glutamic acid, failing to bind with calcium ions. Due to the absence of structural integrity, PIVKA-II was unable to play roles in the activation of coagulation. It was not proposed as an efficient serum biomarker for HCC diagnosis until 1984 (37). Liebman et al. reported that PIVKA-II was significantly increased in 91% of HCC patients without normal sufficient coagulant activity (38). And the number of Gla residues and their positions determined the biological activity of the tumor (39, 40). If the number is less than 5, the liver disease has a higher tendency to be malignant (41, 42). The functions caused by different position deletion in the initiation and progression of HCC need further validations. Numerous studies evaluated its values in predicting prognosis-associated pathological parameters and assessing treatment efficacy in HCC. Recently, PIVKA-II has shown great prospects in other tumor detection and prediction such as primary gastric cancer, pancreatic adenocarcinoma, etc (43, 44).

### Signaling Pathways of PIVKA-II

**Figure 2:**
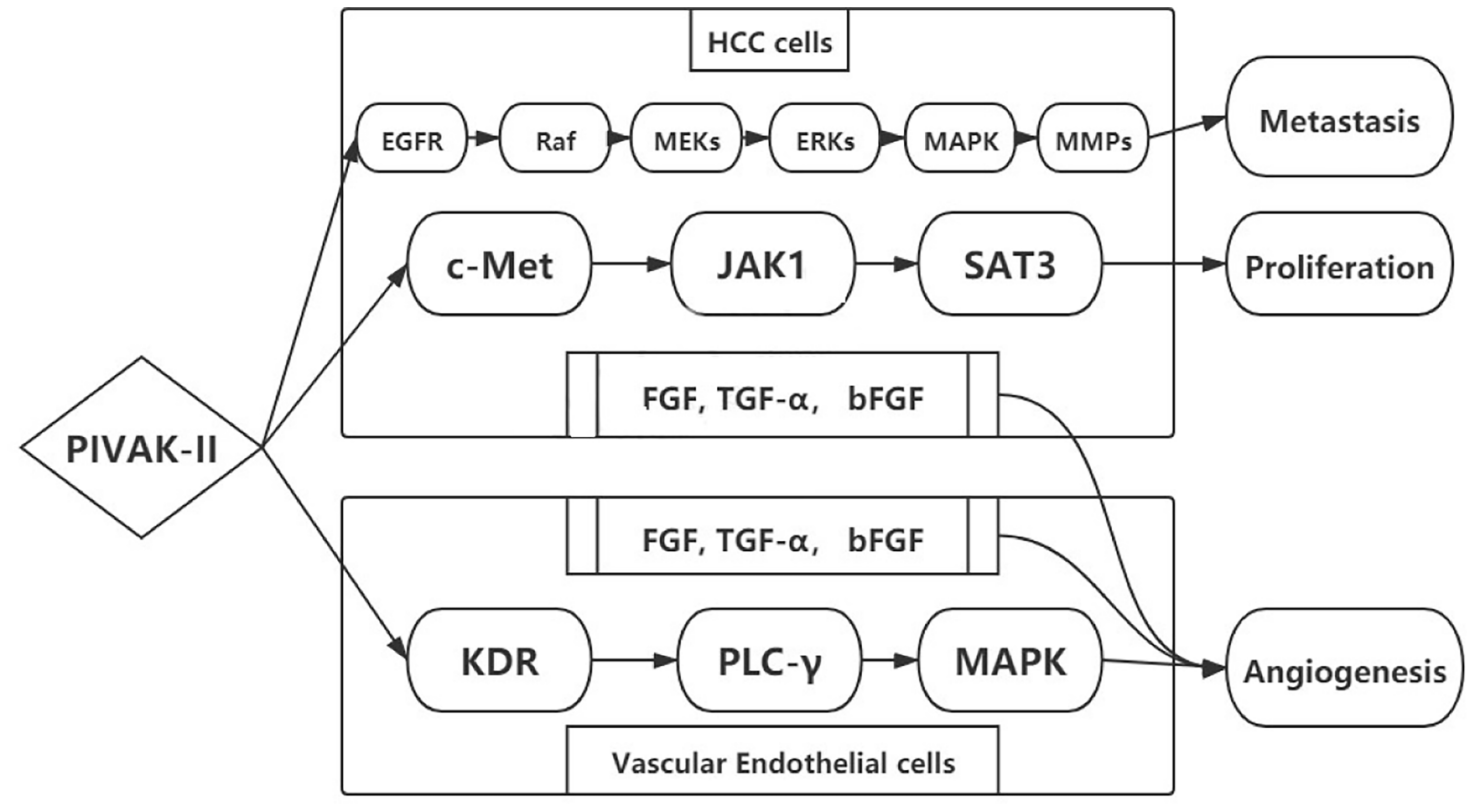
Mechanism of PIVKA-II in HCC progression.

PIVKA-II was a potential autologous growth stimulator for HCC proliferation. The biological process was enhanced by regulating cell proliferation, extracellular matrix (ECM) synthesis, and angiogenesis. Hepatocyte growth factor (HGF) was associated with HCC biological process binding to membrane-spanning receptor tyrosine kinase (c-Met). PIVKA-II could also bind to c-Met by phosphorylation similar to hepatocyte growth factor (HGF) (45). Then the elevated conjugants activated the downstream signals including JAK1-signal transducers and activators of transcription (STAT3), extracellular signal-regulated kinase (ERK1/2)–mitogen-activated protein kinase (MAPK), and kinase insert domain receptor–phospholipase C-γ–mitogen-activated protein kinase (KDR–PLC-γ–MAPK). The activated signaling pathways promoted HCC progression in terms of proliferation, metastasis, and invasion (46–48) (**Figure 2**). The key signaling pathway for HCC proliferation was c-Met-JAK-STAT3 (49, 50). The signaling pathway was activated through the overexpression of PIVKA-II/c-Met that enhanced the DNA synthesis and cell proliferation in HCC (51–53).

Elevated PIVKA-II was associated with bad tumor behavior in terms of metastasis and invasion, leading to a poor prognosis. Many studies have attempted to clarify the mechanism. From current evidence, PIVKA-II could stimulate EGFR phosphorylation, activating Raf serine/threonine kinases. Perhaps PIVKA-II induced the Raf- MEK 1/2 -ERK1/2-MAPK signaling pathway. Then MMP-2 and MMP-9 increased in the context of regulation. With the degradation of the extracellular matrix, MMPs facilitated endothelial cells for matrigel invasion. In addition, the signaling pathway promoted the mitotic process by stimulating mitogens, growth factors, and cytokines (54). This process played a great role on proliferation, metastasis, and invasion in HCC (52).

Angiogenesis was associated with the continuous growth of tumor tissues and tumor progression. Angiogenic factors such as fibroblast growth factor (FGF), transforming growth factor-α (TGF-α), and basic fibroblast growth factor (bFGF) stimulated proliferation and endothelial cell invasion. PIVKA-II was also considered an angiogenic factor. PIVKA-II not only promoted the secretion of other angiogenic factors in HCC cells but also induced KDR–PLC-γ–MAPK signaling pathway in vascular endothelial cells, leading to extracellular matrix degradation and cell migration (55, 56).

## Clinical Relevance of PIVKA-II in HCC

### Surveillance For HCC in High-Risk Population

Surveillance in high-risk populations is an effective measure for HCC early detection. Chronic viral hepatitis-related HCC accounts for 80% of HCC. Patients with virus-related cirrhosis were recommended for HCC surveillance every 6 months (57). Recent studies indicated that patients with other chronic liver diseases such as alcoholic liver disease, non-alcoholic fatty liver disease (NAFLD), and non-alcoholic steatohepatitis (NASH) could also benefit from surveillance (58, 59). A 6-monthly abdominal ultrasonography examination and AFP level measurement are widely implemented for HCC surveillance. The US is an essential first imaging modality, a noninvasive tool despite the sensitivity being 63% (60). Serum biomarker AFP was available in HCC surveillance. However, the levels of AFP were normal in 35–40% HCC patients, causing false negatives. Thus, EASL excluded AFP measurement from the guideline due to its insufficient diagnostic accuracy (61, 62). In this context, serum biomarker PIVKA-II was emerging as a complementary tool for HCC surveillance. Currently, the measurement of combined PIVKA-II and AFP was endorsed in Japan Clinical Practice Guidelines for HCC surveillance (63).

Regarding PIVKA-II as a biomarker, it was promising for HCC surveillance. A meta-analysis evaluated the efficacy of PIVKA-II in 459 cases with hepatitis B virus from June 2016 to March 2018 (64). The pooled sensitivity, specificity, and area under the curve (AUC) were 0.71, 0.93, and 0.91, respectively (64). The study demonstrated the better performance of PIVKA-II in hepatitis B virus-related HCC surveillance. However, due to potential underlying diseases, PIVKA-II alone was not on the point of implementation.

Compared to traditional tools for HCC surveillance, combined PIVKA-II and AFP enhanced the accuracy. PIVKA-II appeared to be more effective in the differential diagnosis between regenerative/dysplastic and neoplastic nodules. A study evaluated the value of HCC surveillance in 90 patients with cirrhotic liver nodules. The result demonstrated that PIVKA-II combined with AFP increased the specificity to 94%, while it decreased the sensitivity. PIVKA-II was more likely to reflect molecular alterations preceding tumor development for cirrhotic patients before imaging discovery. Thus, the combination of PIVKA-II and AFP was a highly promising tool for cirrhotic patient surveillance. Caviglia et al. compared the efficacy of AFP, PIVKA-II, and glypican-3 (GPC-3) for HCC surveillance. The study consisted of 200 patients with viral hepatitis-related cirrhosis and a follow-up period of 36 months. A total of 86 patients who developed HCC had elevated PIVKA-II compared to those still HCC-free. PIVKA-II was the only serum biomarker that could indicate the increased risk of HCC in cirrhotic patients (65).

Considering available repeated peripheral blood samples, changes of PIVKA-II could be applied to personalized dynamic monitoring (66, 67). In 2020, a case-control study assessed the levels of PIVKA-II in patients with cirrhosis during the 12-month observation period. The results showed that PIVKA-II had an increasing trend over time during the progress to HCC. The value at the latest time-point in HCC-developed patients and HCC-free patients were 66 and 32 mAU/ml, respectively (p<0.001) (68). The study suggested that PIVKA-II could serve as an alternative and dynamic tool for HCC surveillance and made it possible to tailor personalized surveillance (64).

### Early Detection in HCC

Given the difficulty in early detection in HCC, only 20–30% of asymptomatic patients had the chance of radical resection (69). Serum PIVKA-II has received much attention as a biomarker for earlier detection to achieve long-term survival in HCC. The well-established biomarker AFP evaluated in many studies had high proportions of false positives. The novel biomarker PIVKA-II had better diagnostic performance with higher specificity in HCC early detection despite the conditions including vitamin K deficiency, malnutrition, drug (e.g., warfarin), and obstructive jaundice (64, 70, 71). In addition, the mechanism of PIVKA-II and AFP was independent. The concentrations of two biomarkers in the same patient were unrelated to each other (72). In this context, PIVKA-II was under investigation as a promising complementary tool (73, 74).

Multiple studies supported that the combination of PIVKA-II and AFP had better diagnostic efficacy in HCC with considerable 67% sensitivity and 100% specificity (75, 76) (**Table 1**). Hemken et al. reported that the levels of AFP and PIVKA-II both elevated in patients with HCC, while the combination was superior to any biomarker alone in early detection (area under the curve 0.85, 95% CI 0.81–0.88) (80). Such findings were similar in a validated study (81). In the study, a diagnostic nomogram based on biomarkers for patients with chronic liver diseases has been constructed to improve diagnostic accuracy. The reliability was confirmed by a multicenter validation study. The combination of AFP and PIVKA-II had good performance in HCC early detection especially for differential diagnosis [AUC, 0.922 (95% CI, 0.908–0.935), sensitivity 88.3%, and specificity 85.1% for the training cohort; 0.902 (95% CI, 0.875–0.929), sensitivity 87.8%, and specificity 81.0%, for the validation cohort] (81). Recently, studies described the GALAD score based on gender, age, AFP, AFP-L3, and PIVKA-II. The score had the superior efficacy (sensitivity 93.3%, specificity 85.6%) regardless of imaging findings (78, 82). Indeed, PIVKA-II could be considered a complementary biomarker to others for early detection in HCC.

**Table 1:** Studies of diagnostic performance of PIVKA-II and its combinations in HCC.

Author	Year	N	Biomarkers	Cut-off value	Diagnostic Performance		
					Sensitivity (%)	Specificity (%)	AUROC
Yu R. et al. (77)	2016	45/183	PIVKA-II+AFP	PIVKA-II =32mAU/ml AFP= 5.0 ng/ml	88.0	85.2	0.886
Lim TS et al. (78)	2016	361/637	PIVKA-II+AFP+AFP-L3	PIVKA-II=40mAU/ml AFP=20ng/mL AFP-L3 = 5%	87	60.1	0.877
Sultanik P. et al. (79)	2017	46/162	PIVKA-II	PIVKA-II=73mAU/ml	74	85	0.89
			PIVKA-II+AFP	PIVKA-II=128mAU/ml AFP=20ng/mL	87	76	A
Loglio A. et al. (76)	2020	64/212	PIVKA-II	PIVKA-II=48mAU/ml	64	91	0,776
			PIVKA-II+AFP	PIVKA-II=48mAU/ml AFP=4.2ng/mL	80	86	0.830
Caviglia G. et al. (65)	2020	149/349	PIVKA-II	PIVKA-II=73mAU/ml	68	84	0.790
			PIVKA-II+AFP	PIVKA-II=73mAU/ml AFP=9.7ng/mL	70	86	0.822
Feng H. et al. (70)	2021	168/321	PIVKA-II	PIVKA-II=35.6mAU/ml	83.93	91.50	0.90
			PIVKA-II+AFP	PIVKA-II=35.6mAU/ml AFP=17.76ng/mL	87.50	92.50	0.94

However, whether combined biomarkers could improve the accuracy was still a point of contention. In 2020, contradictory results were described that combined PIVKA-II and AFP was not better at identifying HCC at the early stage (83).

A needle biopsy was the golden standard for confirmed diagnosis in HCC. Its widespread use was restricted due to the risk of complications such as bleeding, infection, and needle tract seeding (84). Imaging examinations were minimally invasive and effective. However, it depended on the expertise of the physician and technological devices, resulting in difficulties in confirmed diagnosis (85). Affordable, minimally invasive, convenient, and efficient serum biomarker PIVKA-II was highly anticipated as a complementary tool to liquid biopsy for confirmed diagnosis in HCC (77, 86, 87).

### Assessment of Response to Therapy in HCC

Currently, available treatments for HCC included hepatectomy, transarterial chemoembolization (TACE), radiofrequency ablation (RFA), liver transplantation, targeted therapy, and immunotherapy (88, 89). With the advancements of HCC therapeutic strategies and growing awareness of molecular pathogenesis, accurate treatment evaluation based on a molecular level was crucial for selecting optimal treatment strategy. PIVKA-II was considered to have an influence on the assessment of response to therapy for HCC patients in two ways.

On one hand, PIVKA-II may help in preoperative evaluation. PIVKA-II levels identified good candidates who will benefit from liver transplants (LTs). Current Milan criteria (MC) for LT were defined as a single tumor ≤5 cm or the tumor number was less than 3, tumor size ≤ 3 cm, and no major vascular invasion. The criteria were so strict that fewer patients gain. Expended criteria based on serum biomarkers have been proposed. Studies reported that preoperative elevated PIVKA-II was an independent predictor of recurrence and metastasis after LT. Patients with levels of AFP+PIVKA-II ≤ 300 mAU/ml could benefit from liver transplant. In addition, a meta-analysis confirmed that elevated PIVKA-II was associated with a fivefold increased risk for HCC recurrence after liver transplant (90–92).

Compared to hepatic resection, radiofrequency ablation (RFA) had the following advantages: minimal invasiveness, definite efficacy, high safety, and fast recovery. However, when serum levels of PIVKA-II>100 mAU/ml, patients who received hepatic resection had a better prognosis compared to patients with local ablation therapy (24). He et al. established a scoring system for predicting tumor recurrence after RFA. The scoring system suggested that PIVKA-II>40 mAU/ml was an independent risk factor of early recurrence after RFA in HCC (93, 94). In this context, PIVKA-II could provide useful information to improve outcome prediction for HCC management.

On the other hand, early changes of PIVKA-II levels were detected as an indicator for monitoring response to treatments in HCC. PIVKA-II contributed to establishing a standard for assessing the efficacy of hepatectomy. Kim et al. evaluated the trends of PIVKA-II in 184 patients with curative surgery. They found that 94 patients (51.1%) with elevated PIVKA-II levels postoperatively developed intrahepatic recurrence during the follow-up period. Results showed that postoperative PIVKA-II>40 mAU/m indicated poor therapeutic efficacy of hepatectomy (95). The assessment would also apply to TACE. Reduced PIVKA-II after TACE indicated good efficacy. Levels of PIVKA-II in HCC patients with TACE were evaluated. Patients with no PIVKA-II response had shorter survival (7.1 *vs* 75.8 months) and a high risk of cancer-related death (P = 0.02) (95). Another study suggested that advanced patients with a reduction of PIVKA-II >50% after TACE had better survival compared to patients with no response (67.0 *vs*. 19.8 months) (96, 97). Similar findings were subsequently observed in patients with curative ablation that postoperatively elevated PIVKA-II levels were associated with poor prognosis (24). Overall, the measurement of serum PIVKA-II should be integrated into clinical practice for response assessment.

### Prognostic Prediction in HCC

HCC has a poor prognosis with a high degree of malignancy. The overall survival varies from 3 to over 60 months (98, 99). The prognosis was associated with clinical staging in terms of tumor burden (number and size) and vascular invasion, which were independent prognostic risk factors for HCC patients (79). However, current staging systems for prognostic prediction in HCC were based on imaging or pathologic results and rarely included serum biomarkers. Serum biomarkers AFP and PIVKA-II have been approved as predictors for HCC prognosis. A novel staging system including remnant liver function (such as bilirubin and albumin) and serum biomarkers (such as PIVKA-II, AFP, and AFP-L3) had been established to predict survival regardless of the diverse etiology of HCC. The system showed good prognostic prediction efficacy with real-time and dynamic monitoring (100–103).

The preoperative macrovascular invasion could be detected by imaging examination, while microvascular invasion (MVI) relied on postoperative pathological results. How to detect MVI preoperatively is constantly in question. As an angiogenic factor, serum PIVKA-II was proposed as a predictor for MVI in HCC. It was not clear what the optimal cutoff was. Many investigators have assessed the cutoff values (**Table 2**) (24, 104–107). Poté et al. represented PIVKA-II 90 mAU/ml as an independent predictor of MVI (HR 3.5; 95% CI 1.08–11.8; p=0.043) (109). Ryu et al. reported that the cutoff value was 55 mAU/ml (OR, 5.50; 95% CI, 2.09–15.4; P< 0.001) (107). In addition, serum PIVKA-II could be used as a supplement to imaging findings in the prediction of macrovascular invasion. Wu et al. evaluated preoperative levels of PIVKA-II and postoperative pathological results through 31 patients with macrovascular invasion. Results suggested that PIVKA-II>166 mAU/ml was a significant predictor of macrovascular invasion (OR 2.997; 95% CI, 1.217–7.381; P=0.017) in HCC (106).

**Table 2:** Cutoff value of PIVKA-II as an independent predictor in HCC vascular invasion.

Author	Year	N	Cut-off Value	OR	P Value
Masuda T. et al. (24)	2016	46/217	100mAU/ml	2.61	P = 0.02
Wang X. et al. (104)	2017	14/59	32mAU/ml	1.003	P = 0.047
Okamura Y. et al. (105)	2018	76/425	55mAU/ml	9.74	P < 0.001
Wu J. et al. (106)	2018	31/91	166mAU/ml	2.997	P = 0.017
Ryu T. et al. (107)	2019	49/111	55mAU/ml	5.50	P < 0.001

Elevated PIVKA-II was associated with aggressive clinicopathological features. Although total tumor burden may be associated with high levels of PIVKA-II, some studies reported that HCC tumors with elevated PIVKA-II had a higher malignancy grade and were potentially more prone to metastasize (110, 111). Suk-Won Suh et al. reported that the level of PIVKA-II≥800 mAU/ml could predict micrometastases and poor prognosis (HR=5.166; 95% CI, 1.031–25.897) (112). In addition, serum PIVKA-II levels were related to tumor size, tumor cell differentiation, and BCLC staging (P <0.05) (108). Si et al. reported that the serum levels of PIVKA-II were significantly different in well, moderate, or poor differentiation HCC (108).

Patients with elevated PIVKA-II were characterized by a high relapse rate and short overall survival. Early and aggressive recurrence occurred after curative therapies (113). Elevated PIVKA-II may reflect the potential of HCC progression regardless of optimal treatment. According to previous literature, serum levels of PIVKA-II>217 mAu/ml was an independent risk factor of early HCC recurrence (p = 0.0001) (114, 115). Feng Gao et al. reported that the cutoff value of PIVKA-II was 445 mAU/ml (RR=2.307, 95% CI: 1.132–4.703, P=0.021) (33). However, the association between low PIVKA-II levels and longer survival was weak (116). Since liver dysfunction affected prognosis, a multicenter study investigated disease-free survival and overall survival of HCC patients with Child-Pugh classes B and C. Patients with PIVKA-II<90 mAu/ml had better 5-year survival. Elevated PIVKA-II levels were the superior prognostic predictor for HCC recurrence (117).

## Conclusions and Future Prospective

PIVKA-II was a prothrombin precursor produced in HCC. The coagulation function was insufficient due to the lack of Gla residues. Liebman et al. first described PIVKA-II as a biomarker for HCC diagnosis in 1984. Much evidence indicated that elevated PIVKA-II in HCC was associated with malignant tumor behavior in terms of proliferation, migration, and angiogenesis. Three major signaling pathways were proposed to clarify the mechanism. The key signaling pathway for HCC growth was PIVKA-II-c-Met-JAK-STAT3. Raf-MEK1/2-ERK1/2-MAPK-MMPs were associated with HCC migration and invasion. PIVKA-II was considered as an angiogenic factor through KDR–PLC-γ–MAPK, highly expressed in both HCC cells and vascular endothelial cells. The improved understanding of PIVKA-II at the molecular level might provide potential druggable targets for HCC treatment (118).

HCC is a malignant tumor with high morbidity and mortality. Since radical resection remains the only potentially curative treatment for HCC, early detection is essential. Over the past decades, US and biomarker AFP have been widely used for HCC management with suboptimal accuracy. Given the difficulties in early detection, patients with HCC had poor prognosis. To prolong survival, serum biomarker PIVKA-II was proposed for early detection and prognostic prediction in HCC.

PIVKA-II was a potential serum biomarker for HCC. With the advantages including affordability, minimal invasiveness, convenience, and efficiency, PIVKA-II could improve HCC management in four aspects. First, PIVKA-II was an effective and dynamic tool for improving HCC surveillance in high-risk populations. Changes of serum levels of PIVKA-II offered valuable molecular alteration information before imaging discovery. Second, PIVKA-II offered a complementary approach for HCC early detection. Compared to traditional diagnostic approaches, the combination of PIVKA-II and other biomarkers had better performance. Third, PIVKA-II was an indicator for the assessment of response to treatment in HCC. Preoperative assessment was for selecting personalized therapy, and postoperative measurement was for assessing treatment efficacy. Fourth, PIVKA-II was considered as a prognostic predictor for HCC. Patients with elevated PIVKA-II were more likely to develop microvascular invasion, metastasis, and recurrence.

Despite the progressions of PIVKA-II in HCC, some points need to be addressed. The first is whether PIVKA-II associated with other biomarkers could significantly improve the efficacy for HCC management. Second, what is the optimal cutoff value? Third, what could be the mechanism of PIVKA-II in HCC progression? Further studies are required.

Although serum PIVKA-II was not widely accepted in clinical practice, it is highly anticipated as a biomarker for surveillance, early detection, treatment monitoring, and prognostic prediction in HCC.

## Author Contributions

All authors helped to perform the research; YY and JL were involved in the conception and design; GL assisted in searching the latest literature about the role and application of PIVKA-II; JK assisted in language polishing; ZL assisted in creation and modification of figures and charts; YY and YL were involved in the drafting of the paper or revising it critically for intellectual content; JL were involved in the final approval of the version to be published. All authors agreed to be accountable for all aspects of the work.

## Funding

This work was fund by the National Natural Science Foundation of China (General Program), No.81770646.

## Conflict of Interest

The authors declare that the research was conducted in the absence of any commercial or financial relationships that could be construed as a potential conflict of interest.

## Publisher’s Note

All claims expressed in this article are solely those of the authors and do not necessarily represent those of their affiliated organizations, or those of the publisher, the editors and the reviewers. Any product that may be evaluated in this article, or claim that may be made by its manufacturer, is not guaranteed or endorsed by the publisher.
